# Suicide in the Early Stage of Schizophrenia

**DOI:** 10.3389/fpsyt.2016.00116

**Published:** 2016-06-27

**Authors:** Antonio Ventriglio, Alessandro Gentile, Iris Bonfitto, Eleonora Stella, Massimo Mari, Luca Steardo, Antonello Bellomo

**Affiliations:** ^1^Department of Clinical and Experimental Medicine, University of Foggia, Foggia, Italy; ^2^Department of Mental Health, Regione Marche, ASUR, Area Vasta 2, Jesi, Italy; ^3^Department of Mental Health, Regione Veneto, ULSS 13, Mirano, Italy; ^4^Department of Psychiatry, University of Naples SUN, Naples, Italy

**Keywords:** suicide, suicidal attempts, first episode of psychosis, schizophrenia

## Abstract

Suicide is a relevant leading cause of death among patients affected by schizophrenia. Even if suicidal ideation may be present in different stages of disease, some differences have been described between the risk of suicide in patients experiencing first episode of psychosis and those with long-term schizophrenia. It is particularly higher during the first year of illness and reaches a steady decline over the following years. Suicidal ideation and attempts may also be common among subjects with subthreshold psychotic experiences. Factors associated with the risk of suicide in the early phase of schizophrenia are previous suicidal attempts and social aspects: the lack of social support and stable relationships, social drift after the first episode, and social impairment. Also, several psychotic symptoms (suspiciousness, paranoid delusions, mental disintegration and agitation, negative symptoms, depression and hopelessness, and command hallucinations) and substance abuse are associated with higher risk of suicide. It has been described that perfectionism and good levels of insight among individuals who have recently developed psychotic symptoms are significantly associated with higher numbers of suicidal attempts. Moreover, recent evidences show that prefrontal cortex-based circuit dysfunction may be related to suicide in the early stage of schizophrenia. This narrative review summarizes available evidences on suicide in the early stage of schizophrenia and deals with issues to be further studied and discussed.

## Introduction

The risk of suicide can be valued in a multistage continuum, which includes suicidal ideation (ideation, intent, and plans), as a critical initial step, attempted, and completed suicide. The long-term suicide risk in subjects without mental disorders is 0.3%, whereas the risk measured among mentally ill patients ranges from 3.4% for people affected by one mental disorder to 6.2% for people reporting more than one psychiatric disorder: each additional psychiatric diagnosis seems to contribute significantly to increase risk of suicide (1). Suicidal ideation, obviously, is a predictor of suicide and the basis of suicide prevention in schizophrenia (2). Also, suicide is a relevant leading cause of death among patients affected by schizophrenia spectrum disorders, and the rate of attempted suicide in psychotic patients ranges from 10 to 50% (3, 4). Individuals affected by schizophrenia (40–79%) have had suicidal ideation at least one time during the course of illness (5, 6). Also, in schizophrenia, the estimated suicide rate is 579/100,000 person years and the lifetime risk of suicidal death is 5.6% (7, 8). Anyway, rates of completed and attempted suicides among schizophrenia patients are lower than those reported for patients affected by other psychiatric conditions: 0.24 and 0.74 per 100 person years, respectively (9). In particular, the suicide-related mortality is higher among subjects recently diagnosed with schizophrenia (≤5 years from diagnosis) (9). In fact, the suicide risk is twofold higher at the onset of psychotic illness than in the later course (8, 10, 11). The first episode of psychosis (FEP) can be divided in four phases: (a) prodromic phase or emerging psychosis, (b) untreated psychosis (UP) (the duration of untreated psychosis is labeled as DUP), (c) acute psychosis and its treatment, and (d) post-psychotic recovery. Each phase is characterized by different risk of suicide as summarized in the Table 1. In the first phase (that may be called “at-risk mental state” or “prodromic”), suicidal behaviors may be due to the distress caused by unfamiliar emerging pre-psychotic experiences. The delay in accessing the mental health-care system and starting treatment (called DUP) may greatly contribute to increase suicide risk among schizophrenia patients at FEP (12). During the acute phase of schizophrenia, psychotic experiences (distressing delusions, command hallucinations, or passivity phenomena) and feelings, such as fear, stigma, and loss (in patients with some degree of insight), are relevant factors for suicide. Risk of suicide during the following phase of post-psychotic recovery may be related to the loss of role and function mostly due to neurocognitive sequelae (13).

**Table 1:** **Risk of suicide during the first psychosis episode (FEP) phases**.

Phase	Incidence/epidemiology	Possible risk factors
Prodromic phase or emerging psychosis	About 90% of the young people meeting criteria for an at-risk mental state report suicidal ideation (91)	Distress caused by unfamiliar emerging pre-psychotic experiences
Untreated psychosis (UP-phase) and duration of untreated psychosis (DUP)	Most of patients report suicidal risk during this phase, and 25% have already attempted suicide before seeing a psychiatrist. Rate of completed suicides during the UP is very high (92)	The average delay in accessing health-care system during this phase is 1 year. Suicidality is higher when DUP is longer
Acute psychosis and its treatment (phase)	11% of suicide attempts in the FEP are associated with hallucinations, fear, shame, stigma, guilt, loss, rejection, and despair (76)	Patient’s hallucinations, fear, shame, stigma, guilt, loss, rejection, and despair
Post-psychotic recovery (phase)	After an acute episode, 15% of the patients experience high suicidality for the following 18 months (93–96)	Even if the symptoms of psychosis may remit in this phase, neurocognitive deficits may have an impact on studying, working, and recreational activities

## Method

This narrative review provides an overview on the links between schizophrenia, FEP, and suicide. We selected and commented significant articles published on the topic during the last decades (from 1997 to 2016) searching through PubMed, Cochrane Library, and Web of Science/Web and Google Scholar. Key words employed for research were “First Psychotic Episode and Suicide,” “Schizophrenia and Suicide,” “FEP and Suicide,” “FEP,” “Suicide,” “Schizophrenia self-injury,” and “Schizophrenia and self-harm.”

## Incidence of Suicide in FEP

A British 10-year follow-up study showed that subjects affected by FEP have died from unnatural causes more than general population (OR: 13). Suicide was recognized to be the cause of these deaths, and most of the suicides occurred in the first 2 years (14). According to the available evidences on rates of suicide in first-admission psychotic patients, 23% of these have already attempted suicide, and 15% attempted it before the hospitalization (15). Recently, some authors aimed to test the progress in the field of suicide prevention and described the change in a 20-year period of suicide risk among FEP schizophrenia patients belonging to the same catchment area. It was found that suicide risk decreased over the two decades with a reduction of suicide rate from 11.0 to 2.4% (4, 11). These data may suggest that early intervention and therapy improve the suicidality among FEP patients, even if they may attempt suicide before their contact with any mental health service: in fact, mortality rates may be underestimated because of complete suicides committed before seeking professional help (8). In addition, the prevalence of deliberate self-harm (DSH) behaviors before psychosis is about 18.4% (16).

## Subthreshold Psychotic Experiences and Suicide in Schizophrenia

In the last decades, the research highlighted the importance of early intervention of FEP in order to reduce the DUP. Longer DUP, in fact, is associated with poor outcome in schizophrenia with higher prevalence of suicidal behaviors (17). A Norwegian study showed that, in the early phases of FEP, 38.8% of patients reported suicidal ideation and 25.9% attempted suicide before any treatment (17).

Authors from the *Bonn School* suggested the classification of “basic symptoms” of psychosis, which are subtle, subclinical, and detectable at an early stage such as distressing self-experienced disturbances in perception, thinking, memory, motility, mood, sense of awareness, and mastering (18, 19). Authors underline the importance of detecting these symptoms in the early stage of psychosis to reduce the period of untreated illness (DUI).

A new psychopathological framework called attenuated psychosis (APS) disorder concerning subthreshold psychotic symptoms in youths was included in the Section 3 of the Diagnostic and Statistical Manual of Mental Disorders, 5th Edition, as a new putative disorder. The criteria specify that symptoms of APS are sufficiently distressing to call for clinical attention and very helpful to identify young people at risk for psychosis.

The early stages of psychosis were also labeled as “ultra high risk (UHR),” “at risk mental state (ARMS),” and “clinical high risk (CHR).” It is well known that self-harm behaviors and suicidality are highly prevalent in the UHR population, with rates similar to those observed in samples diagnosed with psychotic disorders (20). In addition, about 50% of young people recognized to be CHR for psychosis have reported current suicidal thoughts (21, 22). The prevalence of suicidal ideation among these samples is 42.9%, and there may also be less severe parasuicidal ideation (22).

A meta-analysis of factors associated with DSH shows that the pooled proportion of patients who reported DSH before any treatment for FEP, during the period of UP, and during the period of clinical follow-up between 1 and 7 years from the clinical diagnosis, was, respectively, 18.4, 9.8, and 11.4% (23).

Psychotic-like experiences (PLEs) are common in the general population and may be associated with poor social outcomes, even if there is no diagnosed psychotic disorder (24). Also, they are important but under-recognized markers of risk for severe psychopathology, including multimorbidity, poor functioning, and suicidal behaviors in young people attending the mental health-care services: in particular, individuals with subthreshold psychotic experiences are at increased risk for suicidal thoughts and behavior, similar to those with schizophrenia and other psychotic disorders, but it is unclear whether the level of risk varies with different types of PLEs (25). Moreover, self-reported auditory hallucinations are associated with twofold risk of suicidal ideation and suicidal plans and fourfold risk of suicide attempts in a non-clinical sample of young adults compared with the general population (26). Hypomania, thought control, paranoia, strange experience, and auditory hallucination in subject without definite psychosis were significantly associated with higher suicidal ideation and suicide attempt (ORs ranging from 3.13; 95% CI 1.99–4.93 to 4.03; 95% CI 1.56–10.42) (27). Furthermore, recent studies demonstrated that only a lifetime history of perceptual abnormalities and persecutory ideation are associated with a higher risk of lifetime suicidality, instead, bizarre experiences were not associated with any suicide variable (28). In conclusion, all-cause mortality is associated with lifetime psychotic experiences among schizophrenia patients over a 24- to 27-year follow-up period (after adjustment for sociodemographic characteristics and psychiatric diagnoses), and suicide seems to report a particularly high hazard ratio (9.16, 95% CI 3.19–26.29) (24).

## Risk Factors

Several studies have been carried out on predictors of suicide in patients with schizophrenia in order to improve early detection of suicide risk and to provide suggestions for prevention (11).

### Age of Onset

The relationship between suicide risk and age of onset of psychotic symptoms is complex. In a recent study conducted in a psychiatric unit for adolescents, most of teenagers who attempted suicide were diagnosed with schizophrenia spectrum disorder (29). Some studies have reported a higher risk of suicide in patients with earlier age of psychotic onset (30–32), whereas some other authors have found a relationship with later age of onset of psychosis (33–36): in all studies, age of onset is considered the only independent risk factor for suicide, with risk of suicide increasing by 1.1% per year (4, 33, 35, 36). Individuals who experience a later onset of psychosis may find it more difficult to accept since psychosis may impair their previous functioning, plans, and careers (4). Also, patients with a later age of onset in Singapore reported lower risk of suicide probably due to the fact that young people mostly live with parents throughout the years of schooling, and the family environment can be a protective factor against the risk of suicide (34). In addition, it is of interest that several other studies failed in finding any relationship between suicide risk and age of onset of psychotic symptoms (37, 38).

### Duration of Untreated Psychosis

As stated above, longer DUP is one of the most commonly reported risk factors of poor outcome and suicide among schizophrenia patients (34). Melle et al. (12) showed that patients coming from a community without an early detection program of FEP had a higher risk of suicide if compared with those coming from “early detection communities” and concluded that early detection reduces the DUP and the rate of suicidal behaviors. They also suggested that early engagement of patients in a treatment program seems to have an independent effect on suicidality beyond any DUP (12). Clarke et al. (39) confirmed that individuals with a longer DUP may have a poorer outcome of illness that is associated with higher suicidality. Moreover, in a recent observational study based on a 10-year period of observation, the association between time for remission from psychosis and mortality was studied and found to be statistically significant when adjusted for age at baseline and sex: authors point out that mortality is not only related to DUP but also to other demographic (e.g., sex and age), personal, and outcome factors (including time for remission), and mortality for “unnatural death” may include not only suicides but also accidents (40).

### Gender

Contradictory gender patterns in suicidal behaviors among patients with FEP have been found. Some of these showed no relation with suicide risk, suggesting that the severity of a clinical condition could “override” gender differences in suicidal behaviors (4, 22, 34). In some studies, males seem to be more suicidal than females (41–43), whereas some other reports show that female patients at FEP may show higher risk of suicide and suicidal behaviors (12, 30, 32).

### Living Situation

Studies show that only 20% of the patients who attempted suicide report a comfortable living situation; in most of the cases, there may be a concern regarding loneliness: that may suggest that living with others may be a protecting factor (4). In addition, some authors found an increased risk for suicide in patients who experienced the fear of losing their partner or social position (44). In fact, it is clinically relevant to focus psychotherapeutic interventions on the feeling of loss. It is of interest that the risk of unnatural-cause mortality is reduced by 90% when there is a family involvement at intake or first contact with mental health-care services (14). Further researches are also needed because family involvement and family cohesion are relevant factors that impacts on outcome of illness as well as socioeconomic status, level of education, etc. (14).

It would also be very helpful to integrate and include families and caregivers in the early intervention programs in order to improve the outcome of FEP (40).

### Cognition and Education

Several evidences report that higher education or higher cognitive functioning is associated with an increased risk of suicide in FEP (2, 4, 36, 45, 46). In particular, some of these studies described the relationship between neurocognitive variables and suicidality in patients with schizophrenia spectrum disorders. Nangle et al. have tested suicide attempters and non-attempters with an extensive neuropsychological battery examining premorbid and current general cognitive functioning, episodic memory, and executive functioning. They found that attempters have higher cognitive functioning than non-attempters. Specifically, higher levels of executive functions may influence the ability to plan suicidal behaviors (31). This is in agreement with previous researches showing that higher cognitive functions, in particular, attention and psychomotor speed, verbal fluency, verbal memory, working memory, and executive function, are associated with greater suicidality (47). Nevertheless, recently Barrett et al. have shown that among patients with schizophrenia spectrum disorders, there are no significant differences in neurocognitive functioning between suicide attempters and non-attempters (17). Even if neural dysfunctions responsible for suicide risk are still obscure, some authors have proposed that the prefrontal cortex (PFC) is involved in suicide based on neuroimaging and post-mortem studies. In particular, PFC activity during the goal representation (an important competency of cognitive control) seems to be related to long-term suicide risk in recent-onset schizophrenia, and suicidal behaviors may derive from impairments in premotor cortex support of action planning as an expression of control (48).

Moreover, Björkenstam et al. found a higher risk of suicide among patients who had completed compulsory school. Some other evidences showed that poorer school performance seems to increase the risk of suicide in the general population (49, 50). This may suggest that patients with higher education may feel more stigmatized and shameful when developing a mental disorder (44) and that this may lead to higher risk of suicide (50).

### Psychotic Symptoms in FEP and Suicide

It has been inconsistently shown that negative symptoms may increase suicidal experience in CHR for psychosis individuals (4, 5, 22, 51). Some other authors point out that patients with prominent negative symptoms, in particular, deficits in emotion expressivity, may have significantly impaired ability to experience emotional distress caused by the illness: this may probably reduce the likelihood of developing a sense of hopelessness and suicidal ideation (2). Also, negative symptoms commonly overlap with depressive symptoms, and it is clinically relevant to distinguish the relationship between suicidal ideation and negative symptoms and/or depression among FEP patients. Gill et al. found that negative symptoms remained significantly correlated with severity and intensity of recent suicidal ideation even if adjusted for depression scores (22).

Disorganized symptoms seem to report a poor association with a higher risk of suicide (4). Finally, there is no relevant evidence on the impact of positive symptoms of psychosis and excitement on the risk of suicide (4, 22, 37, 38) even if some studies describe an association between command hallucinations and committed suicides (34, 52). In addition, some authors found that individuals with suicidal ideation during the prodromal phase of schizophrenia report higher scores of negative and positive symptoms than individuals without prodromal suicidal ideation (22, 53).

### Affective Symptoms in FEP and Suicide

Depressive symptoms in the prodromal phase of schizophrenia were frequently associated with suicidality during the following 12 months of outcome (54). In particular, depressive symptoms are associated with lifetime as well as current risk for suicidal behaviors (32, 55) with higher rates of depression after the first episode and any relapse of psychosis (56, 57).

Many authors point out that, in FEP patients, depression and suicidal behavior may be a reaction to the perceived persecutors and entrapment (58). Some other authors found that hopelessness was associated with suicidal ideation in FEP individuals and this symptom predicted suicidal ideation (2).

It has also been hypothesized that suicidality in FEP may be linked to patients’ altered basic self-awareness or sense of self, called self-disorders: there is a clear association between current suicidality and self-disorders, which appears to be connected by depressive states (59). Previously, Skodlar et al. (6) and Skodlar and Parnas (60) suggested that the effect of self-disorders on FEP was connected to specific feelings of inferiority and loneliness, and these feelings were different from “usual” feelings of low self-esteem or loneliness, since they are characterized by being profoundly dissimilar to other people.

“Mood variability” may also be associated with levels of suicidal thoughts and behaviors among individuals at UHR of developing psychosis through the re-activation of latent suicidal cognitions. In fact, in 2012, Palmier-Claus et al. showed that the variability of negative and positive affect was predictive of the frequency of suicidal thoughts and behaviors, with more variable negative affect associated with severe suicidal ideation and related behaviors. In a later study, they also investigated variability and levels of depressive mood, anxiety, and guilty during the schizophrenia FEP and after this episode. The findings support the hypothesis that variability in depression may contribute to suicidal ideation and related behaviors (61).

Early intervention on depression in FEP is crucial to minimize suicidal ideation and attempts, particularly, in the first years of illness, which seem to be consistently characterized by high risk of suicide (2).

Finally, it has been shown that ARMS and FEP patients had significantly higher scores at the brief psychiatric rating scale-excited component (BPRS-EC) if compared with healthy controls: this may suggest an agitated–aggressive syndrome characterized by impulsivity and increased risk of aggression and suicidality (62).

### Schizophrenia and Affective Disorders: The “Continuum” Model

Suicide may significantly occur in the outcome of affective disorders and schizophrenia. Epidemiological data show that unipolar depression, bipolar disorders, and schizophrenia are associated with significant risk of suicide (63). This finding might be explained through the “*continuum*” model in which some characteristics, dimensions, or syndromes (including suicide) are included in different clinical conditions caused by the same underlying mechanism. In fact, several studies proposed a psychopathological *continuum* between schizophrenia and mood disorders (64). In particular, neurobiological data show a relevant overlap between bipolar disorders with psychotic features and schizophrenia. Also, in the early stage of both disorders, genetic vulnerability markers seem to be located on the same chromosomes (65). In addition, schizophrenia and affective disorders present similarities in neurodysfunctions and neuromorphometric characteristics (66, 67). According to this model, risk of suicide may be influenced by different dimensions, such as mood variability and psychoticism, along a spectrum of affective and psychotic disorders.

### History of Suicide Attempts

History of suicide attempts suggests an increased risk of suicide since it is supposed to be a strong predictor of later attempted or completed suicides (2, 9, 68).

Similarly, history of self-harm or violent crime is a relevant risk factor for subsequent suicide in patients with FEP: both include some degree of impulsivity, which is associated with an increased risk of suicide (69).

### Functioning

It is notable that schizophrenia is associated with a significant impairment in occupational functioning that can start early in the prodromal phase of illness.

Also, individuals with recent suicide ideation have poorer functioning, in particular, they report role deficits and lower scores at global assessment of functioning (GAF) (22, 34, 53).

It has been found that social drift is common in psychotic disorders and individuals with a FEP. They are more likely to be in the lower social classes if compared with the general population. Also, social drift was associated with depression, hopelessness, and suicidality at first presentation of illness. However, the relationship between social class and prognosis is complex: hopelessness may be developed in subjects who maintain their social class or achieve upward social mobility. In addition, individuals who achieve upward social mobility are more likely to be ambitious, hardworking, and motivated, and they may have greater difficulty in coping strategies when their life progression is stopped by a psychotic disorder (70).

### Insight

Insight is defined as the awareness of suffering from a mental disorder and needing of treatments. Several studies showed that better insight is associated with suicidal ideation and attempted suicide in FEP patients (71–75), while others specify that insight may influence the risk of suicide if associated with depression and hopelessness (30, 47, 76–79). Conversely, some authors remarked that interventions aimed to improve the insight may also improve the outcome of illness and secondary reduce the risk of attempting suicide (79). In fact, interestingly, it was found that insight at baseline increases the risk for suicide while a good level of insight at 1-year follow-up (due to psychoeducational interventions) decreases the same risk: this may indicate that early insight is qualitatively different from insight after 1 year of treatment. Early insight may imply a negative change in self-image (switching from a healthy person to an ill one) or the awareness of consequences related to a mental disorder and stigma (80). Insight is associated with better treatment adherence in the long term and has a positive impact on the outcome of illness and risk of suicide (81).

In conclusion, authors point out that some domains of insight may increase the risk of suicide such as awareness of mental illness, and also some other factors like being female, longer DUP, and comorbid depression may increase the association between suicide and insight levels.

### Trauma

There are few studies on trauma in FEP and its negative impact on clinical outcome. It is well known that traumatic life events may lead to anxiety, depression, and psychotic symptoms and can contribute to the development of an at-risk state for psychosis (82).

Conus et al. described the prevalence of stressful events in 658 FEP outpatients and their associations with premorbid characteristics, baseline, and outcome differences among subjects who did and did not report past sexual and/or physical abuse (SPA). They found that 83% of these patients had been exposed to at least one stressful event during their lifetime and 34% of them to physical and/or sexual abuse (especially females). SPA patients were more likely to report post-traumatic stress disorder (PTSD) and substance use disorder before the onset of the psychosis to have attempted suicide in the past and during the treatment (83).

The effects of trauma and comorbid PTSD may add further risk of suicide in FEP patients. Tarrier et al. (77) investigated all post-traumatic stress symptoms and the effect on suicidal behavior of trauma associated with the psychotic onset. Eighty percent of the patients felt traumatized, and 38% reported criteria for PTSD. Suicidal ideation was reported by 40% of the sample, and 31% reported attempted suicides. Suicidal behavior rates were higher in those suffering from PTSD, even if not statistically significant and significantly associated with the experience of trauma occurred before the onset of psychosis (77).

These results suggest that treatment of early psychosis must consider childhood trauma and comorbid PTSD. Assessment for PTSD has also been suggested in the National Institute for Clinical Excellence (NICE) guidelines in 2014 since it is helpful for further interventions in FEP patients (84).

### Other Factors

Other factors may also predict the risk of suicide, although the findings are still inconsistent. Patients who completed suicide within 2 years from FEP showed more passive coping strategies or high level of neuroticism (4). Also, perfectionist self-presentation was associated with suicide in a case report of a FEP patient (85).

Family history of a first-degree relative hospitalized for schizophrenia or bipolar disorder or substance use disorder or other mental disorders was supposed to be associated with risk of suicide in FEP patients (69). Illicit drug use was associated with a twofold to fourfold increased risk of all- and unnatural-cause mortality, respectively, while controlling for age and sex (40, 86). The relatively high prevalence of concurrent and lifetime substance abuse in the FEP population may increase the rate of suicidal and aggressive behaviors (87).

Medication history is also to be considered among factors influencing suicide risk among schizophrenia patients. Even if evidences about the efficacy of antipsychotics on suicide and FEP are few, there is an agreement on clozapine and its superiority in treating resistant psychotic illness: in fact, clozapine is associated with a significant 3.3-fold lower overall suicidal risk compared with other antipsychotic treatments (63). Also, in December 2002, the Food and Drug Administration (FDA) approved indication for clozapine to reduce the risk of recurrent suicidal behavior in patients with schizophrenia or schizoaffective disorder (63).

## Limitations

Limitations of this review may include the non-systematic approach in selecting the available literature: even if evidences reported were considered as relevant by the authors, any score assessment of the screened results, as well as any consensus statement among authors were employed. Subjectivity in selecting articles may be considered as a selection bias.

## Conclusion

Suicide is one of the leading causes of premature death among individuals with schizophrenia and psychotic spectrum disorders, and the rate of attempted suicide in these patients is also high (88). This overview confirms that, in recent years, there has been a growing interest in the area of FEP. The most relevant risk factors for suicide in FEP are age of onset of psychotic symptoms, DUP, demographic characteristics, psychopathology, trauma, and insight (a list of risk and protective factors for suicide during FEP is shown in Table 2). Clinicians should assess risk of suicide in the prodromal phase with subthreshold symptoms as well as during the FEP and along the entire course of the illness (89). A more exhaustive monitoring with systematic risk assessment should be conducted regularly during these phases to facilitate early detection of high-risk cases and to provide interventions for suicide prevention without any delay (86, 97). Antipsychotic treatment remains crucial for reducing suicide among FEP patients (90). Clozapine has shown superiority in reducing suicide risk among schizophrenia patients (63). Further studies are required to identify specific psychotherapeutic and psychosocial interventions that may offer more benefits for the prevention of suicidal behaviors in such patients. Specialized multidisciplinary early psychosis teams (psychiatrist, psychotherapist, social worker, etc.) could provide the interventions needed for supporting FEP patients and their family in a comprehensive manner.

**Table 2:** **Risk and protective factors for suicide in FEP patients**.

Risk factors	Protective factors
Acute psychotic symptoms/experiences (e.g., hallucinations)	At least one close relationship
Mood variability and depression	Family support
Pre-existing or comorbid conditions, such as personality disorder and substance abuse/dependence	Things to live for, e.g., plans for the future, children, pets, etc.
The individual reaction to the impact of the illness	Strong positive cultural/religious/personal values and anti-suicide attitudes
Traumatic life events	Social stability
PTSD features related to earlier trauma or prior suicide attempt	Good service engagement and optimism about recovery (hope)
Trauma associated with an unsatisfactory pathway to care	Compliance to treatments
Lower insight	Good insight
Longer DUP	Shorter DUP

*PTSD, post-traumatic stress disorder; DUP, duration of untreated psychosis*.

## Author Contributions

AV, IB, AG, and ES reviewed the literature and drafted the paper. MM, LS, and AB finalized the paper and provided suggestions to improve it.

## Conflict of Interest Statement

The authors declare that the research was conducted in the absence of any commercial or financial relationships that could be construed as a potential conflict of interest.
